# Effect of Daily Physical Activity on Sleep Characteristics in Children with Autism Spectrum Disorder

**DOI:** 10.3390/sports9070091

**Published:** 2021-06-24

**Authors:** Thai Duy Nguyen, Michel Guinot, Véronique-Aurélie Bricout

**Affiliations:** HP2, Inserm U1300, CHU Grenoble Alpes, Univerité Grenoble Alpes, 38000 Grenoble, France; thainguyenduy@hotmail.com (T.D.N.); mguinot@chu-grenoble.fr (M.G.)

**Keywords:** autism spectrum disorders, sleep disorder, physical activity, cluster analysis

## Abstract

**Background**. Sleep problems have been reported in children with autism spectrum disorder (ASD). One of the methods proposed to improve sleep characteristics is based on physical activity (PA). **Objective**. To assess characteristics of sleep and the effect of PA level on sleep quality in children with ASD compared to controls. **Methods**. Fifty boys with ASD (ASD; 10.8 ± 2.6 years) and 18 controls (CONT, 10.1 ± 2.2 years) wore an accelerometer device for five consecutive days to obtain measures of activity and sleep characteristics. **Results**. Some significant differences were reported between ASD and CONT groups. Wake-up time resistance was significantly higher (*p* < 0.05) in ASD. Total time for PA, and daily steps number were significantly lower in the ASD group (*p* < 0.05). Time for sedentary behavior was significantly higher (*p* < 0.01) in the ASD group. Using a principal component analysis and an agglomerative hierarchical analysis, we observed three clusters. Two showed the same poor-quality indices of sleep but two opposing profiles of PA, either an insufficient PA volume (cluster 1, Total time PA = 75.1 min; Daily steps: 7704) or an important PA volume (cluster 3, Total time PA = 222.1 min; Daily steps: 17,164). Cluster 2 was characterized by moderate participation in PA and children with the best sleep indices (Total time PA = 166.8 min; Daily steps: 12,718). **Conclusion**. The dose–response effect of exercise on sleep may indicate large individual differences but the present findings are important to prevent sedentary behaviors or to correct over-activity that could be detrimental to the sleep quality in children with ASD.

## 1. Introduction

Autism spectrum disorders (ASD) is a neurodevelopmental disorder characterized by core clinical features such as social communication difficulties and restricted, as well as repetitive behaviors [1]. Sleep problems are a prominent feature in children with ASD because they lead to deleterious impacts on day-to-day life, and behavioral, emotional, and academic achievement [2]. Sleep plays an important role in both the physical and intellectual development of all children. It helps to improve concentration, cognition and memory [2] and it contributes to the processes of homeostasis [3]. Maintaining the sleep quality of children is thus vital for high levels of mental and physical performance, and for general well-being.

Sleep disorders are one of the most common comorbidities reported in ASD children, occurring in up to 80% of children with ASD compared to 20–40% of typically developing children [4,5,6] including insomnia, circadian rhythm disturbances [5], difficulty falling asleep, restless sleep and frequent wakening [7]. Most comparison studies of sleep based on objective (actigraphy, polysomnography) or subjective (questionnaires) measures have shown poorer sleep parameters in children with ASD than their typically developing peers [4]. Sleep education, environmental changes, behavioral interventions, and exogenous melatonin medication are proposed for promoting and improving sleep quality in children with ASD [8]. Physical activity (PA) is also known to be an effective, non-pharmacological approach and recommended as an alternative or complementary to existing therapies in improving sleep efficiency [9,10,11]. Active participation in PA helps to increase total energy expenditure, and to improve neuropsychological performance and sleep quality indexes [9,12]. Relationships have frequently been found between sleep and physical activity, with positive effects on health, mood, behavior and mental health [3]. Several studies have investigated the effects of exercise in children and adults with ASD. They show clear evidence that exercise improves attention, concentration and organizational skills [2,13].

Nevertheless, children with ASD have fewer opportunities to practice PA because of impairments in their social interactions and communication skills [13,14], even though the PA benefits on their behavioral problems have been demonstrated [2,13]. The contribution of PA in improving sleep quality and cognition among children with ASD has shown its benefits in various studies [11,15,16].

However, many children with ASD (50%) are not engaged in PA with moderate to vigorous intensity for at least 60 min per day as recommended and are characterized by sedentary behaviors [9,12]. Other studies have also indicated that children with ASD have an inactive lifestyle compared to their typically developing peers [15]. These children were less motivated to participate in PA, which resulted in an increased risk of sedentary behaviors, overweight or obesity [16]. Besides, there are many barriers such as individual, social, and community ones that make PA participation more difficult for children with ASD [17]. However, improved PA is an important challenge of concern for children with ASD. Regular PA supports their motor development, improves their physical fitness, and stimulates their social skills and behavior.

The relationship between sleep and PA is still not fully understood and controlled. Patterns of sleep and PA suggest that being physically more active tends to lead to healthier sleep in children with ASD [18]. Some studies have also revealed a significant improvement in sleep efficiency, sleep onset latency, sleep duration, and wake after sleep onset when PA is increased, especially for these children [19]; however, other studies have also reported the deleterious effects of excessive PA on sleep indices [11]. For example, the time of day at which PA is performed could have negative effects on sleep (e.g., the child’s level of excitement might persist and delay falling asleep if PA is less than 4 h before sleep) [11]. Although evidence strongly suggests that PA is beneficial to all children, this is even more significant for children with ASD due to the difficulties that limit their participation in PA [19].

Considering the sleep benefits obtained from participation in PA, it is essential to clearly understand the effects of PA on sleep in this population. In addition, determining what level of PA is appropriate to promote sleep quality in children with ASD is relevant to the successful management of sleep problems. In this context, it has been reported that vigorous PA can reduce sleep onset latency and total sleep time [6]. Thus, PA could have positive impacts on sleep quality by adjusting factors such as level, intensity and duration of the exercise [6,10].

Moreover, the heterogeneous clinical signs that characterize ASD make it difficult to address the management of sleep disorders in children with autism, and the interest to promote PA in this management. In this context, the use of cluster analysis could be an interesting approach to classifying a wide range of information into significant and interesting classes. It is a data reduction tool that creates subgroups that are more understandable than individual variables. It examines the full complement of inter-relationships between variables. Therefore, our study aimed to compare sleep and PA level characteristics in children with ASD to controls, and to assess the effect of PA on sleep quality in children with ASD, with a cluster analysis.

## 2. Methods

This study was approved by the local Ethics Committee of the Hospital (N°A00-865 40), registered on the Clinicaltrials.gov registry (N°CT: 02830022) and was conducted according to the Declaration of Helsinki.

### 2.1. Subjects

Each subject and parent received written and oral information. They agreed to participate and signed a consent form. Sixty-eight boys volunteered to participate in the study: 50 children with ASD (ASD; 10.8 ± 2.6 years; 7 to 17 years) and 18 controls (CONT; 10.1 ± 2.2 years; 7 to 17 years; Table 1), all of them were attending regular schools. The control children were typically developing children recruited from different local schools. They were active children who were free to participate or not in physical activity but were not involved in competitive sports.

Diagnosis of ASD was performed in early childhood by experienced physicians and psychologists, according to the Diagnostic and Statistical Manual of Mental Disorders 5th edition criteria [1]. The subjects were also assessed with the Autism Diagnostic Observation Schedule (ADOS) [20]. Intellectual Quotient (IQ) was estimated using the Wechsler Intelligence Scale for Children, 4th edition [21]. The IQ criterion for the children was IQ > 70, excluding children with intellectual disabilities (IQ < 70). Following the ethical guidelines, IQ scores and ADOS results were not provided to researchers. However, score certifications with IQ > 70 for all ASD children in this study were confirmed by a clinical psychologist with experience in diagnosing children with ASD. All children had an initial medical check-up to verify the absence of infectious diseases and to review any medication the children were taking. Children with psychiatric disorders or comorbid medical, exercise contraindications for exercise and those taking medication were excluded from the study [22]. Initially, 73 boys were recruited but only 68 were retained in this study because 3 CONT and 2 children with ASD failed to obtain results due to their non-cooperation in wearing the accelerometer device.

### 2.2. Actigraphy

Each participant wore the accelerometer monitoring device (Sensewear^®^ Pro Armband 3, Body media, Pittsburgh, PA, USA) for five consecutive weekdays (Monday to Friday). Inclusion criteria for the actigraphy monitoring required that child tolerated the wrist device placement during the study. Participants and their parents completed daily diaries to distinguish periods in which participants did not wear the accelerometer (shower or bath, swimming or other water activities). The analysis did not include those periods. The monitoring device used in this study was a biaxial accelerometer, worn on the right arm triceps. It estimated energy expenditure based on algorithms from measured parameters such as heat flux, galvanic skin response, skin temperature, near-body temperature, and demographic characteristics such as sex, age, height, weight.

This device can measure sleep parameters such as sleep time, sleep latency, wake up time, wake after sleep onset and sleep efficiency. It was also used to calculate the parameters related to PA such as the time for PA with different levels (sedentary, moderate, and vigorous) and energy expenditure for PA. PA with an energy expenditure between 3 and 6 Mets was categorized as moderate, PA > 6 Mets and <9 Mets as vigorous, and PA > 9 Mets as strongly vigorous.

### 2.3. Child Sleep Diary and Questionnaires

*Child sleep diary*: Children and/or their parents recorded information related to sleep each night. This included bedtime (the time when the children went to bed each evening), wake up time (the time when the children woke up each morning), and sleep time (duration sleep time of the children each night based on the parents’ estimation).

*The children’s sleep habits questionnaire* (CSHQ): The CSHQ was developed by Owens et al. [23]. This is the most used questionnaire to evaluate the sleep of children with ASD. CSHQ includes 45 items that are score-rated from 1 to 3 and divided into eight subscales (bedtime resistance, sleep onset delay, sleep duration, sleep anxiety, night waking, parasomnias, sleep-disordered breathing and daytime sleepiness). The CSHQ total score was calculated and compared with the threshold value of 41. If CSHQ total score was higher than 41, it indicated the presence of sleep disorders or low sleep quality.

*The physical activity questionnaire for children* (PAQ-C; [24]): PAQ-C contains 9 items with scores rated from 1 to 5. For this study, it provided general estimations of the level of PA and sedentary activities of the children over the past seven days. The final PAQ-C summary score was calculated to reflect PA levels: Light (score = 1), Moderate (score = 2–4), and Vigorous (score = 5).

## 3. Statistics

The data collected from the monitoring device were extracted by a specialized software (Sensewear Software, Version 7.0, Pittsburgh, PA, USA). The average value of weekdays (WD, Monday to Friday) for each child was calculated (Table 2). Results are given as mean ± standard deviation (SD). The first objective was to compare children with ASD to CONT (with a Student’s *t*-test after having checked the conditions for the suitable application of parametric or non-parametric tests). An effect size (Cohen’s d value) was also calculated and added for each significant result. A d value of around 0.2 is described as a “weak” effect, 0.5 as “medium” and 0.8 as “strong”.

Significance was accepted when the *p*-value was lower than 0.05. The second objective was to assess the effect of PA level on sleep quality in children with ASD. For this purpose, a principal component analysis (PCA) and an agglomerative hierarchical cluster analysis (AHCA) were performed on data obtained by actigraphy (R software^®^, version 4.0). AHCA is a method used to bring all observations into a cluster and build a hierarchy of clusters. The image obtained gives an overall view of the relationship between all the collected data [25]. The PCA was supplied with the normalized version of the original predictors. Here, normalization and centralization of the data by the feature scaling method were first applied [26]. In this part of our study, we had *n* = 50 observations (50 children with ASD) and *p* = 10 predictors (5 predictors related to PA data and 5 predictors related to sleep data from actimetry), therefore respecting the application rule for PCA: 5 observations per 1 predictor. We used the elastic net method, which simultaneously permits automatic selection of pertinent variables. It can also select groups of correlated variables [27]. The 10 predictive variables selected were:

- Total bed time (a1)
- Sleep duration (a2)
- Sleep quality index (3)
- Sleep latency (a5)
- Wake-up time resistance (a6)
- Total time for PA (a13)
- Time for sedentary behavior (a14)
- Time for MVPA (moderate to vigorous PA) (a16)
- Time for vigorous PA (a17)
- Daily steps number (a19).

A factor map was also proposed. This graphical representation shows the partition of our data into different clusters and classifies each child, using different colors, in order to indicate the membership of specific items from different clusters. Based on these observations, an average score was calculated in each cluster, using the following method for each variable: a score of 3 was assigned to the most favorable observation, and a score of 1 to the least favorable value. A subtotal was finally calculated for the sleep and PA variables. With these scores, it was possible to define a label to characterize each cluster (Table 3).

## 4. Results

Demographic data, questionnaire results, sleep and PA variables are presented in Table 1. There was no statistical difference in demographic characteristics between the two groups (Table 1). The PAQ-C and CSHQ results showed that children with ASD had significantly altered scores compared to CONT children with regard to their PA and sleep characteristics (Table 1). In both groups, the questionnaire-estimated sleep duration compared to the sleep duration measured by actigraphy was significantly higher (+2.5 h; *p* < 0.001).

Regarding sleep and PA variables, some significant differences were observed between the ASD and CONT groups. Wake-up time resistance was significantly higher (*p* < 0.05; Table 2) in ASD compared to CONT children. MVPA energy expenditure, total time for PA, time for MVPA, vigorous and strong vigorous PA and number of daily steps were significantly lower in the ASD group (*p* < 0.05; Table 2). Time for sedentary behavior was significantly higher (*p* < 0.01; Table 2) in the ASD group.

**Figure 1:**
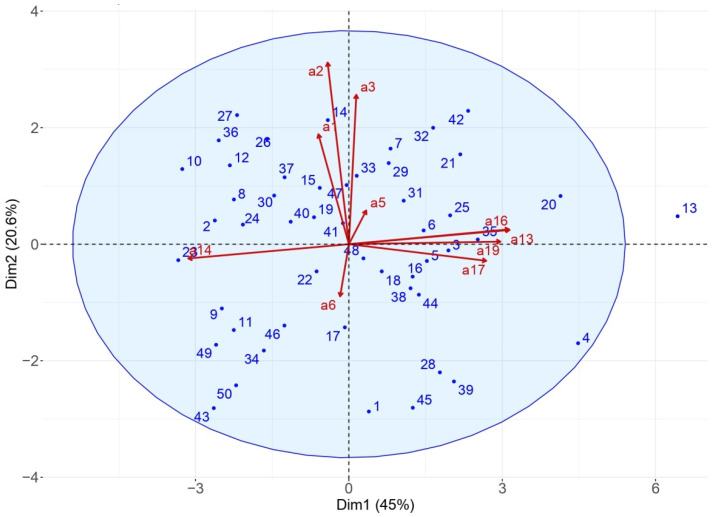
Principal component analysis (PCA) biplot. Dim: dimension. Children are represented from 1 to 50 and 10 predictive variables with the letter a, such as total bed time on (a1); sleep duration (a2); sleep quality index (a3); sleep latency (a5); wake-up time resistance (a6); total time for PA (a13); time for sedentary behavior (a14); time for MVPA (a16); time for vigorous PA (a17); daily steps number (a19).

The PCA was applied to the ASD group, on the ten significant variables that helped explain 65.6% of the variance on the first two dimensions (45% and 20.6%), which was a satisfactory outcome. In the biplot representation (Figure 1), a child on the same side of a given variable obtained the best score for this variable. A low value for this variable was attributed to a child on the opposite side. For example, a child positioned near item a2 (such as child #7) presented a mean sleep duration of 7 h 15, and a child on the opposite side (such as child #45) presented a mean sleep duration of 5 h 38.

The upper left quarter included children with long sleep duration and total time in bed, while the lower right quarter included children with very short sleep duration. In the lower left quarter were the children who had high sedentary behaviors, on average 2 h 50 min higher than children in the upper right quarter.

**Figure 2:**
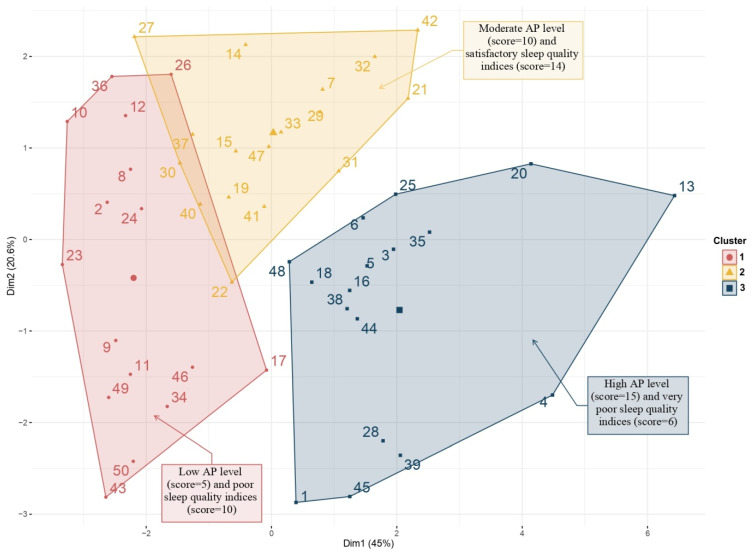
Factor map obtained by agglomerative hierarchical cluster analysis (AHCA) in ASD group.

The agglomerative hierarchical cluster analysis allowed the classification of children based on sleep and PA characteristics. This analysis ranked each child on a factor map, using different colors. On this representation (Figure 2), three clusters were obtained that successfully classified the children into:

- Cluster 1 (pink): A total of sixteen children with ASD were included in this cluster. They were characterized by limited participation in PA with very low values for the variables: MVPA energy expenditure, total time for PA, time for moderate PA, time for vigorous PA, and daily steps number. Also, these children were the most sedentary. They had the highest value for sleep latency, but other sleep characteristics were average.
- Cluster 2 (saffron yellow): A total of seventeen children with ASD were included in this cluster. This cluster was characterized by children with moderate participation in PA and who had the best sleep indices. They also reached the highest score for total bed time, sleep duration, sleep quality and middle score for physical activity.
- Cluster 3 (blue): A total of seventeen children with ASD were included in this cluster. This cluster was characterized by children with active participation in PA and poor-quality sleep. They had the highest value for daily steps number, total time for PA, time for moderate PA, and time for vigorous PA. They had the shortest sleep duration, with significant sleep latency and wake after sleep onset. Their sleep quality index was the lowest of the 3 clusters.

In this graphical representation, one child with ASD (#13) was classified outside of these clusters. The child #13 was characterized by a total of daily steps two times higher than the group average (25,620 vs. 12,354 steps/24 h). He had the highest time for vigorous PA (118 vs. 18 min/24 h in mean), strong vigorous PA (16 vs. 2 min/24 h in mean). Associated with these PA characteristics, he showed altered sleep variables with a sleep duration of 7 h per night, a sleep latency of 19 min (vs. 13.6 ± 9.7 in mean), a bedtime resistance of 18 min (vs. 12.8 ± 7.0 in mean) and an awakening latency of 24 min (vs. 15.5 ± 8.6 in mean).

Representation with the factor map allowed us to specifically localize the position of one subject within its own cluster and the neighboring cluster. Thus, subject #32 was in cluster 2, but some of his characteristics were similar to cluster 1. Subject #30 belonged to cluster 1, but also presented one (or more) of the characteristics of cluster 2. In contrast, none of the children in clusters 1 and 2 had PA or sleep characteristics classified identical to cluster 3.

## 5. Discussion

The present study focused on the sleep and physical activities assessments of children with ASD to evaluate the effect of PA level on sleep quality in children with ASD. As already reported by many authors, our results confirmed that children with ASD showed poor sleep indices related to PA level [11,16,18,19,28,29,30]. The recommended amount of sleep needed for optimal health, as suggested by pediatric experts, is 8 to 10 h of sleep for all children, with or without ASD. Nevertheless, some studies have reported shortened total sleep duration associated with difficulty falling asleep, frequent night-time awakening, and early morning awakening in children with ASD [4,5,7,29]. These sleep disorders occurred in 44% to 83% of children, which represents a major problem for parents [4]. In addition, these children have decreased levels of PA compared to their typically developed peers, even though a number of studies have provided convincing evidence of the benefits of physical activity on health and sleep quality in these two populations [3,26,30]. PA and sleep influence each other through multiple complex interactions in both physiological and psychological processes [3]. PA is usually considered as beneficial for improving sleep quality [31].

Within the scope of this study, the effect of PA level on sleep quality in CONT and children with ASD was evaluated. The comparison between the two groups showed some differences in PA indices. Children with ASD were much less active than CONT children, specifically in moderate-to-vigorous physical activity and tended to show increased sedentary behaviors. This observation was mentioned in recent studies [30,32], but more MVPA and less sedentary behavior were associated with better quality sleep [30]. A previous review reported that the time spent in MVPA of children with ASD was 34–166 min/day (Jones et al., 2017). In our study, our results were precisely in this range (142–154 min/day), but time for MVPA was significantly lower for ASD children compared to CONT (−53 min, *p* < 0.05). However, some studies have showed that for every additional hour of time for MVPA, beneficial effects on sleep have been observed, for example, sleep onset advanced by 18 min, sleep duration increased by 10 min and sleep efficiency by 0.6% [30].

Children with ASD practiced enough PA even though these practices were always significantly less than the ones from CONT children. Many studies have identified the reasons for these low PA values: fewer clubs to practice sports, fewer associations that welcome children, fewer people trained to supervise children in sports clubs, but there are also many parental barriers (economic, temporal and emotional) [19,33,34]. Although children with ASD were practicing less PA than CONT, they were following the World Health Organization recommendations, particularly with regard to MVPA (60 min per day) on weekdays [33]. Moreover, these differences could not be attributed to differences in accelerometer wear time since no significant differences were found between the two groups (Table 2). The children correctly wore the device without any withdrawal or even sensory problem, and the identical values in our two groups confirmed perfect adherence to the study. This device is widely used to assess PA variables for children with ASD [34]. Numerous studies have also shown interest in using this device to better evaluate sleep characteristics because it allows objective measurements. Thus, an exciting finding was observed while comparing the results of the data recorded by the actigraphy and those reported by the parents (questionnaire; Table 1). Parents’ estimated sleep time was always significantly higher than sleep time measured by the accelerometer, with a mean difference of 2.5 h in both groups. This demonstrated that the children did not go to sleep according to the schedule that their parents set. Furthermore, the analysis of the CSHQ questionnaire confirmed that ASD children have significantly impaired sleep compared to CONT. Hering et al. reported significant differences in parental evaluation of night-time awakening, and early morning awakening in children with ASD as compared with typically developing control [35]. These authors stated that “Parental oversensitivity to sleep disturbances of the autistic children may explain this phenomenon”, even though these differences were not found with actimetry assessments.

In ASD, possible effects of PA on sleep have been reported by several authors. PA might have a positive or negative effect on sleep quality [6,11]. It has recognized effects on mental health, by reducing anxiety and depression, which are sources of insomnia [36]. In addition, it promotes thermoregulatory mechanisms, a key element in sleep induction. Indeed, the drop of temperature after PA or during recovery is essential for falling asleep [37].

Therefore, it is essential to understand how PA level can have a positive impact on sleep. The PCA, followed by an AHCA in the ASD group allowed us to classify the children into three clusters in which we observed distinct PA and sleep characteristics. Cluster 1 and cluster 3 showed the same poor-quality of sleep but also two opposing profiles of PA (insufficient PA or important PA). Cluster 2 showed moderate participation in PA and children who have the best sleep indices.

Moderate physical activity and good-quality sleeping habits could conversely be beneficial and trigger a virtuous circle that improves health [38]. Moderate PA and MVPA were reported to enhance sleep quality by decreasing sleep latency, and wake after sleep onset (Master et al., 2019). This suggestion was in line with results reported in a comprehensive review of studies about the effect of PA on sleep quality with different PA intensities [10]. In our study, we observed that insufficient or excessive physical activity had a negative impact on the quality and quantity of sleep. Although sedentary lifestyle has often been reported to explain this sleep alteration, it has less often been established that an excess of PA could be so deleterious [6,10]. Some authors have reported this same observation in a clinical case study [6,11], and this was also confirmed with child #13, who had significant PA but impaired sleep latency, bedtime resistance and awakening latency. These previous studies [6,11] concluded that regular, well-dosed PA was most appropriate to ensure quality of sleep.

In ASD, where sleep disorders are major, this information could be very important. PA is a simple, inexpensive intervention, whose benefits on the core-symptoms of ASD are significant. In cluster 1, we can also observe that the children in this group are the oldest and have the higher BMI. These two factors are known to have an impact on the level of physical activity. The higher the BMI, the lesser PA practiced, and the more sedentary time spent in the PA. Moreover, several studies have shown that the level of PA decreases in adolescents compared to younger children. Age is considered a critical factor affecting PA, with younger age being linked with higher PA [28,39]. On the other hand, sedentary habits, physical activity, self-determined motivation, motor skills, and sleep patterns affects the PA levels of children with ASD [14,26,35]. PA is an important factor for health in populations with autism spectrum disorders, and the benefits of physical activities for children with ASD have been studied [28,33]. It has been found that regular PA helps to improve a number of deficits in children with ASD. In addition, some studies have reported that several of the benefits derived from PA are related to mental and psychosocial wellbeing (cognitive and adaptive abilities).

Finally, our study shows some strengths and limitations. Firstly, in order to increase the statistical power and confidence in the generalizability of the results related to PCA and AHCA, there is a need for higher recruitment of subjects. Secondly, because only boys with IQ > 70 were included in our study, it is necessary to reproduce the same work with children with more severe ASD. Future research should also consider a group with girls, and subgroups examining older or younger children with ASD. The strong points of this work include the use of objective methods of sleep and PA evaluation coupled with questionnaires, and the application of original statistical methods.

## 6. Conclusions

This study confirmed the difference in PA level and sleep characteristics in children with ASD compared to controls. It indicated that ASD children tend to have low participation in PA and more sedentary behaviors than typically developed children. The dose–response effect of PA on sleep characteristics may indicate large individual differences, but with the present classification obtained by relevant methods (PCA and AHCA), it could be possible to inform parents about the type and the volume of PA in order to prevent sedentary behaviors of children with ASD or to reduce their PA to prevent it from becoming deleterious to sleep.

The novelty of our study was that we assessed and classified what level of physical activity may impact sleep characteristics of children with ASD. Classification methods based on PCA and AHCA are not new statistical methods, but it was their use in this ASD population that allowed us to better understand the effects of PA level on children’s sleep and to define three clusters, which was new. The need to classify autism heterogeneity (concept of spectrum) has gained importance with the development of better care of individuals with ASD characteristics. This classification highlighted the disparity in clinical, physiologic, and pathologic markers in ASD.

Physical activity could be used as a non-pharmacological method to improve sleep disorders in ASD children, but the nature and the magnitude of this PA needs to be properly planned. Future studies shall clarify the mechanism of PA level effect on sleep quality.

## Figures and Tables

**Table 1:** Demographic data and characteristics of sleep and PA by questionnaire.

		ASD	CONT	D Value
**Demographic**	Age (years)	10.8 ± 2.6 (7–17)	10.1 ± 2.2 (8–17)	0.07
	Weight (kg)	39.2 ± 15.7 (15.4–90.0)	35.7 ± 13.0 (22.2–79.8)	0.11
	Height (cm)	147.2 ± 16.0 (114–180)	143.8 ± 14.7 (124–188)	0.02
	BMI (kg/m^2^)	17.5 ± 3.8 (11.8–33.3)	16.7 ± 2.1 (13.1–22.6)	1.51
**Survey**	PAQ-C score	2.6 ± 0.7 (1.1–3.8)	3.2 ± 0.6 (2–4.2) **	0.78
	CSHQ score	47.8 ± 6.5 (37–65)	44.4 ± 4.5 (39–52) *	0.47
	Sleep duration (h; questionnaire)	9.5 ± 1.2 (7.5–12)	9.5 ± 1.0 (8.5–10.5)	0.04
	Sleep duration (h; actigraphy)	7.00 ± 0.8 (4.9–8.5) ^££^	7.1 ± 0.7 (5.7–8.3) ^££^	0.05

Values are mean ± SD; (min–max values). ASD: children with autism spectrum disorders; CONT: control; BMI (body mass index); PAQ-C: The physical activity questionnaire for children; CSHQ: The children’s sleep habits questionnaire; h: hours. ^££^ *p* < 0.001: significantly different between sleep duration by questionnaire and actigraphy. * *p* < 0.05; ** *p* < 0.01: significantly different between ASD and CONT: independent t-test significantly different between ASD and CONT.

**Table 2:** Characteristics of sleep and PA by actimetry.

		ASD	CONT	D Value
**Sleep variables**	Total wearing time of actimeter	23.4 ± 0.4	23.0 ± 0.7	0.06
	Total bed time (h)	9.6 ± 0.7	9.9 ± 0.8	0.48
	Sleep duration (h)	7.0 ± 0.9	7.1 ± 0.8	0.12
	Sleep quality index (%)	74.9 ± 7.1	74.4 ± 7.4	0.08
	Bedtime resistance (min)	12.8 ± 7.0	10.5 ± 4.4	0.31
	Sleep latency (min)	13.6 ± 9.7	15.9 ± 6.6	0.21
	Wake-up time resistance (min)	1.4 ± 3.0	0.2 ± 0.4 **	0.44
	Awakening latency (min)	15.5 ± 8.6	19.3 ± 6.7	0.43
	Wakening after sleep onset (min)	113.0 ± 41.0	126.7 ± 49.3	0.31
**Physical activity**	Total energy expenditure (kCal)	1602 ± 566	1650 ± 594	0.07
	MVPA energy expenditure (kCal)	430 ± 237	594 ± 272 *	0.61
	Total time for PA (min)	158 ± 77	216 ± 59 **	0.77
	Time for sedentary behavior (min)	1282 ± 77	1223 ± 61 **	0.77
	Time for MVPA (min)	156 ± 75	209 ± 59 *	0.72
	Time for vigorous PA (min)	20 ± 21	42 ± 19 ***	0.96
	Time for strong vigorous PA (min)	2 ± 3	6 ± 6 *	0.86
	Daily steps number	12,658 ± 4833	15,739 ± 3286 *	0.58

Values are mean ± SD. ASD: children with autism spectrum disorders; CONT: control; h: hours; min: minutes; MVPA: moderate to vigorous physical activity (see methods). * *p* < 0.05; ** *p* < 0.01; *** *p* < 0.001: significantly different between ASD and CONT.

**Table 3:** Average score in each cluster for all variables used in agglomerative hierarchical cluster analysis.

	Cluster 1 (Pink)		Cluster 2 (Yellow)		Cluster 3 (Blue)	
SLEEP VARIABLES	Mean	Score	Mean	Score	Mean	Score
Total bed time on (h)	9.6	2	10.0	3	9.2	1
Sleep duration (h)	6.8	2	7.7	3	6.5	1
Sleep quality index (%)	72.8	2	79.6	3	72.5	1
Sleep latency (min)	15.1	1	11.3	3	14.8	2
Wake-up time resistance (min)	1.1	3	1.4	2	1.8	1
Total score for sleep variables	-	10	-	14	-	6
PA VARIABLES	Mean	Score	Mean	Score	Mean	Score
Total time for PA (min)	75.1	1	166.8	2	222.1	3
Time for sedentary behavior (min)	1365.0	1	1272.6	2	1218.2	3
Time for MVPA (min)	74.6	1	164.8	2	217.6	3
Time for vigorous PA (min)	6.2	1	15.0	2	36.6	3
Daily steps number	7703.8	1	12,718.5	2	17,163.8	3
Total score for PA variables	-	5	-	10	-	15

ASD: children with autism spectrum disorders; CONT: control; h: hours; min: minutes; MVPA: moderate to vigorous physical activity.

## Data Availability

The data presented in this study are available on request from the corresponding author. The data are not publicly available due to medical characteristic of these data that represent privacy data.
